# Trauma-focused therapies for post-traumatic stress in psychosis: study protocol for the RE.PROCESS randomized controlled trial

**DOI:** 10.1186/s13063-022-06808-6

**Published:** 2022-10-05

**Authors:** Simone R. Burger, Tineke van der Linden, Amy Hardy, Paul de Bont, Berber van der Vleugel, Anton B. P. Staring, Carlijn de Roos, Catherine van Zelst, Jennifer D. Gottlieb, Kim T. Mueser, Agnes van Minnen, Ad de Jongh, Machteld Marcelis, Mark van der Gaag, David van den Berg

**Affiliations:** 1 Department of Clinical Psychology, VU University and Amsterdam Public Health Research Institute, Room MF-B543, Van der Boechorstraat 7, Amsterdam, 1081 BT the Netherlands; 2 Department of Psychosis Research and Innovation, Parnassia Psychiatric Institute, The Hague, the Netherlands; 3 Department of Research and Innovation, GGzE Mental Health Institute, Eindhoven, the Netherlands; 4 Institute of Psychiatry, Psychology and Neuroscience, King’s College London, London, UK; 5 South London & Maudsley NHS Foundation Trust, London, UK; 6 GGZ Oost-Brabant Mental Health Institute, Boekel, the Netherlands; 7 GGZ Noord-Holland-Noord Mental Health Institute, Alkmaar, the Netherlands; 8 Altrecht Mental Health Institute, Utrecht, the Netherlands; 9 Academic Centre for Child and Adolescent Psychiatry Level, Amsterdam University Medical Centre (location AMC), Amsterdam, The Netherlands; 10 Cambridge Health Alliance & Harvard Medical School Department of Psychiatry, Cambridge, MA USA; 11 Boston University, Boston, USA; 12 Behavourial Science Institute, Radboud Universiteit Nijmegen, Nijmegen, the Netherlands; 13 PSYTREC Mental Health Institute, Bilthoven, the Netherlands; 14 Department of Behavioral Science, Academic Centre for Dentistry Amsterdam (ACTA), University of Amsterdam and VU University Amsterdam, Amsterdam, the Netherlands; 15 Institute of Health and Society, University of Worcester, Worcester, UK; 16 School of Psychology, Queen’s University, Belfast, Northern Ireland; 17 Faculty of Health, Medicine and Life Sciences, Maastricht University, Maastricht, the Netherlands

**Keywords:** Psychosis, PTSD, Trauma, Trauma-focused treatment, Cognitive restructuring, Prolonged exposure, EMDR therapy

## Abstract

**Introduction:**

Many people with psychotic disorders experience symptoms of post-traumatic stress disorder (PTSD). In recent years, several trauma-focused therapies (TFTs), including cognitive restructuring (CR), prolonged exposure (PE), and eye movement desensitization and reprocessing (EMDR) have been studied and found to be safe and effective in reducing PTSD symptoms in individuals with psychosis. However, studies were conducted in different countries, with varying inclusion criteria, therapy duration, control groups, and trial outcomes. RE.PROCESS will be the first study to compare the impact of CR, PE, and EMDR with a waiting list control condition within the same context.

**Methods and analysis:**

This is the protocol of a pragmatic, single-blind, multicentre, superiority randomized controlled trial, in which CR, PE, and EMDR are compared to a waiting list control condition for TFT (WL) in a naturalistic treatment setting. Inclusion criteria are as follows: age ≥ 16 years; meeting full DSM-5 diagnostic criteria for PTSD on the Clinician-Administered PTSD Scale for DSM-5 (CAPS-5), with a total CAPS score ≥ 23; and a psychotic disorder in the schizophrenia spectrum confirmed by the Structured Clinical Interview for DSM-5 (SCID-5). Participants (*N*=200) will be randomly allocated to 16 sessions of one of the TFTs or WL, in addition to receiving treatment as usual (TAU) for psychosis. The primary objective is to compare the effects of CR, PE, and EMDR to WL on researcher-rated severity of PTSD symptoms over time from baseline to 6-month follow-up. Secondary objectives are to examine these effects at the separate time-points (i.e., mid-treatment, post-treatment, and at 6-month follow-up) and to test the effects for clinician-rated presence of PTSD diagnosis, and self-rated severity of (complex) PTSD symptoms.

**Discussion:**

This is the first RCT to directly compare the effects of CR, PE, and EMDR within the same context to TAU on PTSD symptoms in individuals with psychosis and PTSD. Secondary effects on clinical and functional outcomes will be investigated both directly after therapy and long term.

**Trial registration:**

ISRCTN ISRCTN56150327. Registered 18 June 2019.

**Supplementary Information:**

The online version contains supplementary material available at 10.1186/s13063-022-06808-6.

## Introduction

Exposure to traumatic events is highly prevalent in individuals with psychotic disorders [1]. Experiencing childhood trauma increases the likelihood of developing psychotic symptoms [2, 3] and is associated with persistence of psychosis [4]. Individuals with severe mental illnesses are also at increased risk of revictimization [5, 6]. In individuals with psychosis, post-traumatic stress disorder (PTSD) is a common comorbidity (12.4-16.0%) [7, 8]. PTSD and psychosis symptoms tend to fuel each other, with symptoms interacting and overlapping [9–12].

Several studies have shown trauma-focused therapy (TFT) to be feasible and effective in reducing PTSD symptoms in individuals with psychosis, with more equivocal findings regarding their effects on psychotic symptoms [13]. Three TFTs targeting PTSD have been studied in psychosis with rigorous designs: cognitive restructuring (CR), prolonged exposure (PE), and eye movement desensitization and reprocessing therapy (EMDR). The 16-session CR intervention [14] was found to reduce PTSD symptoms in severe mental illnesses in two separate randomized controlled trials (RCT) in the USA [15, 16], in which a minority of participants had a psychosis diagnosis (15% and 33% respectively). A UK study with participants with psychosis found that while CR did significantly reduce PTSD symptoms, there were also significant reductions for the TAU group [17].

Pilot studies have reported positive effects of PE [18, 19] and EMDR [19, 20] on PTSD symptoms in psychosis samples. A Dutch RCT confirmed these results, comparing the effects of eight sessions of either PE or EMDR to waiting list for TFT (WL) [21, 22]. Both PE and EMDR were associated with a significant decrease of PTSD symptoms, and post-treatment effects were generally maintained at 12-month follow-up [23]. However, since most participants experienced multiple severe childhood traumatic events, the programme length of eight sessions was found to be too brief to address all the intrusive memories for many participants in the study.

Therefore, more treatment sessions could enhance the effects of these interventions, as observed in previous TFT studies [24, 25]. Providing additional sessions would also allow for targeting psychosis-related traumatic events when PTSD symptoms have resolved sufficiently. Psychotic experiences can be linked to traumatic events, often reflected in the content or the appraisal of the psychotic experiences (for example: hearing the voice of a perpetrator or interpreting shadowy figures as a replay of past persecution) suggesting that trauma processing can be beneficial. By targeting psychosis-related traumatic events, we aim to address a broader concept of post-traumatic reactions that includes psychosis [26].

Although CR, PE, and EMDR have similarities, they are based on different theoretical backgrounds and employ different therapeutic techniques [27]. Moreover, the context in which these therapies were tested differed greatly. The studies were executed in different countries, adopted slightly different inclusion and exclusion criteria, focused on targeting psychosis outcomes to varying degrees, and tested different dosages of therapy. Also, expertise of therapists may have differed and factors such as TAU for psychosis or PTSD differ amongst countries, influencing study results. Comparing all three TFTs to WL within the same context will provide more insight into their relative effects, working mechanisms, and acceptability.

### Aims and objectives

This trial aims to test the effects of CR, PE, and EMDR in a sample of participants with psychosis and PTSD. The primary objective is to compare the effects of CR, PE, and EMDR to WL on researcher-rated severity of PTSD symptoms (CAPS-5 total score) over time from baseline to 6-month follow-up. Secondary objectives are to examine these effects for researcher-rated severity of PTSD symptoms at the separate time-points (i.e. mid-treatment, post-treatment, and at 6-month follow-up) and to test the effects (over time and at each time-point) for clinician-rated presence of PTSD diagnosis, and self-rated severity of (complex) PTSD symptoms.

## Methods and design

### Trial design

**Fig. 1:**
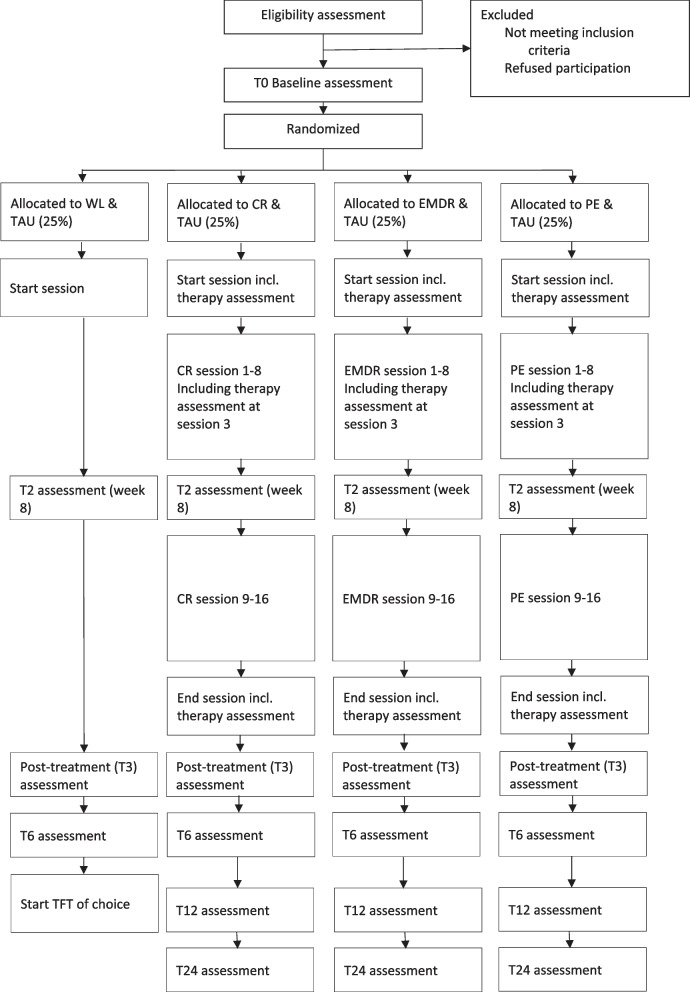
Flowchart of the inclusion, allocation, assessments, and therapy. *Note:* T1 = mid-treatment, T2 = post-treatment, T3 = 6-month follow-up, T4 = 12-month follow-up, T5 = 24-month follow-up

This study is a pragmatic, single-blind multicentre superiority randomized controlled trial with four arms: CR, PE, EMDR, and WL. Therapy in all three active arms will be delivered over 16 sessions by the same group of trained therapists working in routine mental health services to eliminate therapist effects and align as much as possible with the routine clinical reality. All groups receive TAU for psychosis and will be assessed at baseline (T0), mid-treatment (T1) at 7 weeks, post-treatment (T2) at 3 months, and at 6-month follow-up (T3). Participants in the WL condition receive therapy of choice after T3. The CR, PE, and EMDR conditions will also be assessed at 12-month (T4) and 24-month follow-up (T5). Up to the 6-month assessment, social functioning, adversities, and revictimization outcomes will be monitored weekly using smartphone questionnaires to enhance the ecological validity of these measurements. The Consolidated Standards of Reporting Trials (CONSORT) diagram depicting the eligibility and assessment stages is presented in Fig. 1.

### Additional explorations

We plan on the following explorations: (A) examine the effects of the treatments on disruption of social functioning by PTSD symptoms, post-traumatic cognitions, dissociation, depression symptoms, paranoid ideation, presence and impact of auditory hallucinations, sexual functioning, social functioning, personal recovery, adversities, experienced resilience, and revictimization over time and at each time-point; (B) examine long-term effects of the treatments (12- and 24-month follow-up) on all variables; (C) determine the cost-effectiveness and cost-utility of the treatments compared to each other and to WL [28]; (D) test how participants’ expectancy of therapy effects, their perception of the therapist, therapeutic alliance, and treatment effects interact and change over time [29]; (E) test baseline predictors of treatment outcome given that experimental studies on predictors of treatment outcome are scarce [30, 31]; and (F) gain insights into the experience of participants receiving TFT, through qualitative interviews.

### Study setting

Participants are recruited from psychosis outpatient services of multiple mental healthcare organizations in the Netherlands. A list of study sites can be obtained at the ISRCTN registry. All participants receive TAU for psychosis, which consists of care in multidisciplinary assertive outreach teams and usually includes antipsychotic medication, psychological therapies, and supportive counseling by psychologists, caseworkers, nurses, and psychiatrists.

### Eligibility criteria

Inclusion criteria for participants are as follows: (A) aged ≥ 16 years; (B) a psychotic disorder in the schizophrenia spectrum, confirmed by the Structured Clinical Interview for DSM-5 (SCID-5); and (C) meeting DSM-5 symptom criteria for PTSD based on the Clinician-Administered PTSD Scale for DSM-5 (CAPS-5) with a total score ≥23.

Exclusion criteria are as follows: (A) changes in antipsychotic or antidepressant medication within 4 weeks before the eligibility assessment (to control for medication effects); (B) insufficient mastery of the Dutch language; (C) severe intellectual impairment, defined as an estimated IQ of 70 or less; and (D) not willing or able to learn to use a smartphone for weekly assessments.

### Sample size

Sample size for longitudinal intention-to-treat (ITT) analyses with linear mixed models (LMM) was calculated [32] and based on data from previous RCTs [21, 33]. With alpha = 0.05, beta = 0.2, rho = 0.45, and 3 repeated follow-up assessments, and an additional 20% to compensate for dropout, this study needs 50 participants in each arm to detect at least medium effects of 0.5 against waiting list condition. The aim is therefore to randomize 200 participants.

### Interventions

In each therapy arm, participants receive a maximum of 16 therapy sessions, delivered as two 75-min sessions per week. Baseline assessment data of traumatic experiences, PTSD, and psychosis are used to support the development of a case formulation and treatment plan. Treatment and recovery goals are set in a start-session before the beginning of therapy, with progress evaluated and future plans agreed on in an end-session after finishing therapy. All therapies are based on a collaborative therapeutic relationship and entail recognition of the trauma and its consequences, attention, and hope. Participants monitor their post-traumatic stress and psychosis symptoms weekly, and review these with their therapist.

### Cognitive restructuring (CR) for PTSD

CR for PTSD supports people to learn how to manage or modify distressing thoughts. CR for PTDS is based on the cognitive model of PTSD which argues that PTSD symptoms develop and are maintained by unhelpful and inaccurate thoughts and beliefs related to traumatic events, which then manifest in a more global set of distressing and unhealthy beliefs pertaining to the self, others, and the world. The primary focus is on thoughts associated with PTSD symptoms, although thoughts related to any situation can be targeted (including those linked to psychosis), especially in earlier sessions, as the therapist teaches the CR skill to the client so they can move toward mastery. The first 3 sessions of CR teach an anxiety-management skill (breathing retraining) for managing arousal and provide information about PTSD, followed by 13 sessions of CR. Participants learn CR as a simplified, step-by-step self-management skill to deal with any negative feelings and situations. This involves identifying distressing thoughts, evaluating them, and then either modifying them by developing alternatives or making a detailed, systematic action plan to manage distressing them. In CR for PTSD, participants initially learn to cope with any distressing feelings and as their skills develop, shift to trauma-related thoughts and beliefs underlying PTSD symptoms. Home assignments to practice breathing retraining and CR skills are collaboratively set in each session [14].

### Prolonged exposure (PE)

PE is a psychotherapy that aims to reduce PTSD symptom severity by systematically confronting the patient with safe but anxiety-provoking reminders of the trauma, including trauma-related memories (imaginal exposure, including homework of listening to audio recordings) and real-life trauma-related stimuli (in vivo exposure). PE aims to activate the trauma-related fear memory network and change trauma-related cognitions. In the first session, the therapist and the participant develop a case formulation that contains the most important intrusive trauma memories (based on intensity and frequency scores) and the most important avoidance behaviours to which patients are then exposed in the following sessions. PE therapy will be delivered using the Dutch protocol of van Minnen and Arntz [34], based on the protocol of Foa, Hembree, and Rothbaum [35].

### EMDR therapy

EMDR is a psychotherapy that aims to process traumatic memories by simultaneously retrieving the traumatic memory and taxing the working memory. Eye movements will be applied as the default working memory taxation stimulus, with added memory taxation (tapping, spelling words, steps) when eye movements are not sufficient to tax a patient’s working memory [36–38] or not possible due to COVID-19 restrictions. In the first sessions, the therapist and the participant develop a case formulation that contains the most important traumatic experiences, a hierarchy of the most relevant intrusive trauma memories based on their subjective units of distress (SUD), with the memories with the highest SUD being targeted first. EMDR will be delivered according to the standard 8-phase protocol by Shapiro [39], using the Dutch translation [40].

### Therapists

The therapists are trained in all three TFTs (PE, EMDR, and CR), with competence assessed before seeing trial cases. Therapists are frontline clinicians working within the participating institutions, delivering the trial therapies as part of routine care. All therapists are at least Masters level psychologists, with varying experience, training, and expertise in working with psychosis and PTSD. Case formulations and treatment plans are reviewed by an expert supervisor (AH, AM, CR, AJ, TS, PB, BV, TL). Therapists receive monthly group supervision and online on-demand supervision after each session. Therapists provide a fidelity checklist of each session to the supervisor, which the supervisor reviews and provides feedback on. In addition, in line with the CR for PTSD protocol, sessions were rated for competence at regular intervals by supervisors.

### Treatment fidelity

Therapists provide a fidelity checklist of each session to the supervisor. All sessions are videotaped and reviewed in monthly supervision. A random selection of sessions will be rated for treatment fidelity for each TFT. After each session, the therapists fill in a fidelity checklist which is reviewed by a supervisor.

### Procedures

#### Recruitment, baseline assessment, and randomization

Trial therapists and their teams identify participants, screen for initial eligibility using patient records and routine outcome measures, and conduct Module B and C of the SCID-5 on psychotic symptoms to check whether a psychotic disorder is present [41], and then refer them to the study coordinator (SB). The participant receives both oral and written information about the study from the study coordinator or a research assistant and has at least 1-week time to decide upon participation. The participant is asked to sign informed consent and is assessed for inclusion criteria. After the baseline assessment, the participant is randomized to one of the 4 conditions. Randomization is stratified by trial therapist and conducted by an independent researcher via a randomization programme (http://www.randomizer.org). The allocation sequence is saved in a protected folder to which the research team has no access. The independent researcher informs the therapist of the allocation who in turn informs the participant.

### Assessments

**Fig. 2:**
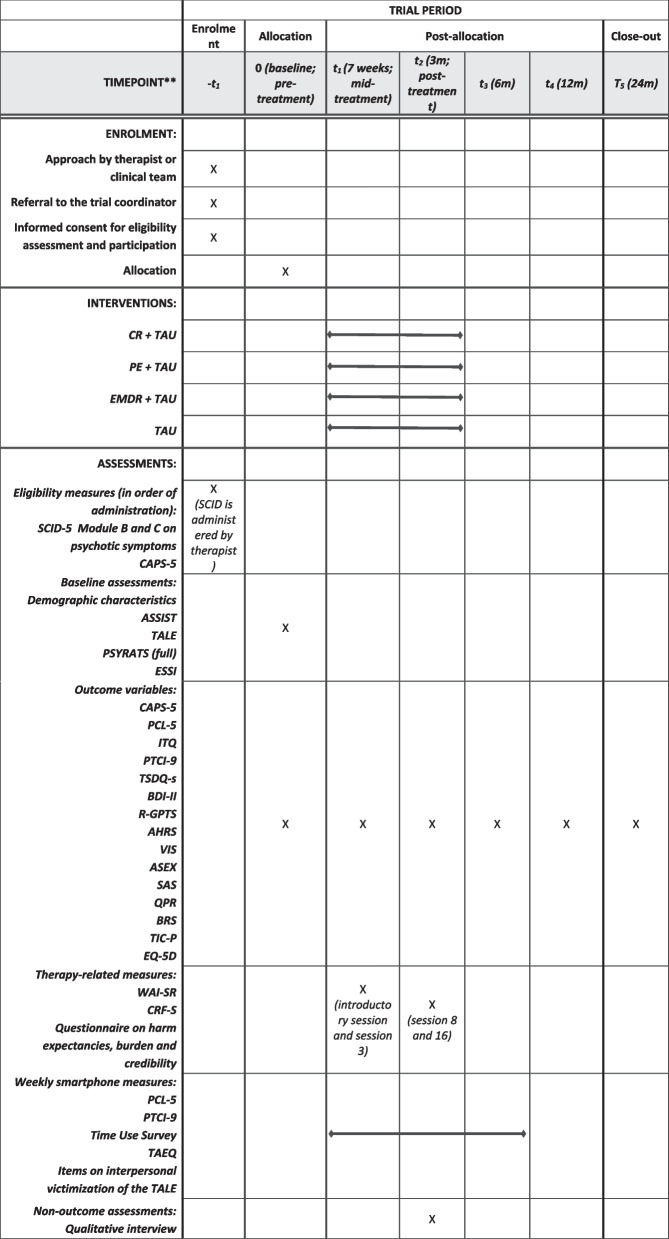
Schedule of enrolment, interventions, and assessments. Structured Clinical Interview for DSM-5 (SCID-5) [41] Module B and C on psychotic symptoms. Clinician Administered PTSD scale for DSM-5 (CAPS-5) [42]. Brief Version of the Alcohol, Smoking and Substance Involvement Screening Test (ASSIST) [43]. Trauma and Life Events (TALE) [44]. Psychosis Symptoms Rating Scale (PSYRATS) [45, 46]. Enriched Social Support Instrument (ESSI) [47]. The PTSD Checklist for the DSM-5 (PCL-5) [48, 49]. International Trauma Questionnaire (ITQ) [50]. Brief Version of the Posttraumatic Cognitions Inventory (PTCI-9) [51]. Trait State Dissociation Questionnaire – short version (TSDQ-s) [52]. Beck Depression Inventory II (BDI-II) [53]. Revised version of the Green et al Paranoid Thoughts Scale (R-GPTS) [54]. Auditory Hallucination Rating Scale (AHRS) [45, 46]. Voice Impact Scale (VIS). Arizona Sexual Experience Scale (ASEX) [55]. Sexual Autonomy Scale (SAS) [56]. Questionnaire about the Process of Recovery (QPR) [57]. Brief Resilience Scale (BRS) [58]. Treatment Inventory of Costs in Patients with psychiatric disorders (TIC-P) [59, 60]. EuroQol 5-dimensions (EQ-5D) [61]. Working Alliance Inventory – Short Form Revised (WAI-SR) [62]. Counselor Rating Form – Short (CRF-S) [63]

A researcher (SB) or research assistant from the VU/Parnassia, who is blind to the allocation of the participant, carries out the (planning of) the assessments (Fig. 2). They are supervised in administering the CAPS-5 interview by a clinical psychologist with extensive experience in administering the CAPS-5 (DB). Research assistants are trained to competence on the interview outcome measures (CAPS-5 and PSYRATS) before conducting any assessments. Research assistants write a clinical report on the CAPS-5 and PSYRATS of every assessment which is reviewed by SB to insure inter-rater reliability and minimize rater drift. In case of incidental unblinding, a different assessor performs the rest of the assessment. Participants are compensated for expenses (e.g. travel costs) with 20 Euro per assessment.

All weekly assessments are conducted using an online questionnaire application (RoQua). Participants receive a text message with a link through which they can access the assessment.

The therapist will provide the hardcopy questionnaires for the participant’s perception of the therapy and the therapeutic alliance during the introductory session, sessions 3, 8, and 16. To ensure anonymity/confidentiality, participants are asked to return the completed questionnaires in a sealed envelope and are informed that the therapist will not be able to review their answers.

A subgroup of approximately 15 participants [64] is invited to partake in qualitative interviews to assess their experience of the TFT 2 to 6 weeks after the post-treatment assessment. Purposive sampling will be applied to include participants from all treatment arms and treatment drop-outs.

### Outcome measures

Outcome measures are shown in Fig. 2.

### Demographics and baseline assessments

Demographic characteristics include age, sex, gender, country of birth (including that of parents), education, employment, living condition, relationship status, duration of PTSD, duration of psychosis, and duration of contact with mental healthcare. Substance use is assessed with the first two questions of the Alcohol, Smoking and Substance Involvement Screening Test (ASSIST) [43]. Social support is measured with the Enriched Social Support Instrument (ESSI), which is a 5-item measure of social support with good validity and reliability [47]. The Trauma and Life Event Checklist (TALE) is a 21-item checklist for traumatic experiences and life events in people with psychosis [44].

### PTSD symptoms

The Dutch version of the Clinician-Administered PTSD Scale for DSM-5 (CAPS-5) is used to assess the severity of PTSD symptoms, the presence of PTSD diagnosis, and the influence of PTSD symptoms on social functioning. The CAPS is anchored to all reported criterion A events, as identified by the TALE. These can include traumatic events associated with the psychosis symptoms (e.g. a voice threatening to kill the participant). The CAPS-5 assesses the intensity and frequency of all PTSD symptoms and yields a continuous severity score. Symptoms with a severity score ≥2 are considered clinically significant. The CAPS-5 is the gold standard diagnostic interview for PTSD [42, 65]. The DSM-5 version has recently been validated and has good psychometric properties [66], also in Dutch translation [67].

The PTSD Checklist for the DSM-5 (PCL-5) is a 20-item self-report questionnaire on assessing all DSM-5 PTSD symptoms. The DSM-5 version demonstrated strong validity and reliability [48, 49].

The International Trauma Questionnaire (ITQ) is an 18-item self-report questionnaire that assesses the presence of complex PTSD as defined in the ICD-11 and the severity of these symptoms, including two items on how PTSD symptoms influence social functioning [68]. Results on psychometrics were encouraging [50].

The Brief version of the Posttraumatic Cognitions Inventory (PTCI-9) measures trauma-related cognitive distortions about the self, the world and self-blame. This 9-item version is based on the original PTCI [51] and was found to have strong psychometrics [69].

The Trait State Dissociation Questionnaire – short version (TSDQ-s) is a 15-item self-report questionnaire and consists of items from existing dissociation measures (including the Dissociative Experiences Scale [70] and the Peritraumatic Dissociation Scale [71]) and new items measuring aspects of dissociation which were not sufficiently represented in existing scales. The TSDQ-s has been shown to be psychometrically robust [52].

### Other psychiatric symptomatology

The Beck Depression Inventory II (BDI-II) is a widely used 21-item self-report questionnaire for depression [53]. It provides a continuous score for the severity of depression symptoms and has excellent psychometric properties [72, 73].

The Revised Green et al. Paranoid Thought Scales (R-GPTS) is an 18-item self-report questionnaire that assesses ideas of persecution and social reference and was found to have excellent psychometric properties [54].

The psychotic symptoms rating scales (PSYRATS) is an 18-item clinical interview that assesses delusions and auditory hallucinations. The PSYRATS subscales have excellent inter-rater reliability and strong validity [45, 46]. We use a slightly augmented version which also captures type and content of delusions and hallucinations. At follow-up, assessments only the frequency, duration, and disruption items on auditory verbal hallucinations will be conducted.

The Voice Impact Scale (VIS) is a 24-item self-report questionnaire that assesses the impact of hearing voices. A publication concerning the psychometrics is pending.

### Sexual functioning, disruption of social functioning, social functioning, and personal recovery

The 5-item Arizona Sexual Experience Scale (ASEX) is a 5-item self-report questionnaire that assesses sexual dysfunction. The ASEX has high validity and reliability, regardless of availability of a sexual partner and sexual orientation [55].

The Sexual Autonomy Scale (SAS) consists of three self-report items measuring the extent to which individuals feel their sexual behaviours are self-determined with good reliability [56].

Social functioning is assessed with two items adapted from the Time Use Survey on the number of minutes spend with others and spend on pleasant activities [74, 75].

The Questionnaire about the Process of Recovery (QPR) assesses personal recovery in 15 self-reported items and has good psychometric properties [57].

### Adversities, resilience and revictimization

Adversities (e.g. self-harm or hospitalization) are measured using the 7-item TTIP Adverse Events Questionnaire (TAEQ) [76, 77]. Revictimization is assessed using eight interpersonal victimization items from the TALE [44]. Resilience is measured using the Brief Resilience Scale (BRS), a 6-item self-report questionnaire of resilience that has good psychometric properties [58].

### Cost-effectiveness and cost-utility

The Treatment Inventory of Costs in Patients with psychiatric disorders (TiC-P) short version is an interview consisting of 17 items measuring the economic costs and benefits of therapy [59, 60]. It asks individuals for the number of contacts with healthcare providers and for productivity losses. The EuroQol (EQ-5D) is the World Health Organization measurement for health outcome that can be expressed in utilities. Utilities can be combined with cost-effectiveness data into quality-adjusted life years (QALYs) [61, 78].

### Therapy-related measures

The Counselor Rating Form – Short (CRF-S) is a 12-item self-report questionnaire that assesses counselor’s amiability, expertness, and trustworthiness as perceived by the participant. The CRF-S has been shown to be psychometrically robust [63].

The Working Alliance Inventory – Short Form Revised (WAI-SR) is a 12-item self-report questionnaire that assesses the strength of the therapeutic alliance as perceived by the participant and has good psychometric properties [62].

Harm expectancies (e.g. being afraid to “lose control” during a session), burden expectancies, and credibility of the therapy as perceived by the participant are assessed with a 9-item questionnaire developed for the Treating Trauma in Psychosis trial [76].

### Qualitative study

Experience of the TFT will be assessed with a semi-structured interview guided by a topic list constructed by the research team.

### Therapy procedures

In PE and EMDR, when PCL-5 scores for sections B (reliving) and C (avoidance) are both 0 for at least two consecutive sessions and the intrusive trauma memories hierarchy contains no PTSD-related trauma memories with a SUD > 0, therapists can target psychosis-related traumatic memories. PE and EMDR therapies can be completed early after a shared decision-making process between participant and therapist when PTSD is in remission (using criteria above) and no psychosis-related trauma memories or imagery have a SUD > 0. In CR, participants are encouraged to complete all 16 sessions even if PTSD remits prior to this time, to support generalization of learning and planning for the future.

The participant and treatment team are asked not to start any other form of TFT, to refrain from CBT and changes in medications until the 6-month follow-up. However, in the case of an adverse event or an increase in (psychosis) symptoms that demands intervention, antipsychotics, sedatives, or CBT may be increased or provided without consequences for therapy and/or study participation.

### Withdrawal of individual participants

Participants can leave the study at any time for any reason if they wish to do so without any consequences. The investigators can decide to withdraw a participant from the study for urgent medical reasons. When a participant withdraws from treatment and is still willing to partake in assessments, follow-up assessments will be conducted. There will be no replacement of individual participants after withdrawal.

### Analysis

The primary and secondary outcomes will all be analysed on an intention-to-treat basis. We will perform completers (sensitivity) analyses to test the robustness of the outcomes. Both primary and secondary continuous outcomes will be analysed with linear mixed models (LMM) with baseline values included as covariates. Dichotomous primary and secondary outcomes will be analysed with Generalized Estimating Equations (GEE). The main outcome is the effect over time including all data points between baseline and 6-month follow-up. The effects at the different time-points will also be computed using interaction terms.

To test whether the effects of CR, PE, and EMDR on the primary and secondary outcomes endure in the long term, we will test the changes from 6-month follow-up to the 12-month and 24-month follow-up respectively. These within-group changes will be analysed with paired samples *t*-tests (continuous outcomes) and McNemar’s tests (dichotomous outcomes). Although not powered, we will also do an exploratory analysis for differences between CR, PE, and EMDR at these time-points with independent samples *t*-tests and chi-square tests for independence. The predictive value of the potential predictor variables will be tested with multiple linear regression analysis.

If a therapy is found to be effective compared to WL at 6 months, cost-effectiveness and cost-utility analyses will be performed in the same way as in the TTIP trial [28]. The incremental cost-effectiveness ratios (ICERs) are considered a single-point estimate of an underlying continuum. Acceptability curves are produced with bootstrap simulations and confidence intervals. The outcome will be costs in Euro’s per QALY and the costs in Euros per day without PTSD gained. If none of the therapies are effective, which is not expected, a cost-minimization calculation will be performed. For all TFTs, interaction between the participants’ perception of the therapy, view of the therapist, strength of the therapeutic alliance in different points of the therapy, and treatment effects will be studied using regression analysis and Spearman’s rho. Predictive value of the baseline variables will be investigated using regression analyses. Thematic analysis will be used to analyse themes in the interviews with regard to how participants have experienced the TFT [79, 80] consisting of data familiarization, initial coding, searching for themes, reviewing themes, defining and naming themes, and finally producing the report. Analysis will be performed in parallel with conducting the interviews.

### Monitoring

The study was classified as imposing negligible added risk through research procedures on participants by the medical ethics committee of the VU University Medical Centre. Therefore, a trial management group was established in place of a data monitoring committee. No interim analyses will be performed. Serious adverse events (SAEs) are monitored by therapists and supervisors and will be reported to the medical ethics committee.

### Ethics

This trial was set up in accordance with the SPIRIT checklist (Standard Protocol Items for Randomized Trials; Additional file 2) [81, 82]. The study protocol was approved by the Medical Ethics Committee of the VU University Medical Centre (METC number: 2019.046/ NL66431.029.19) and the trial was published in an online registry (ISRCTN56150327). METC and registry are notified of any protocol changes. The study is conducted according to the principles of the Declaration of Helsinki and in accordance with the Dutch Medical Research Involving Human Subjects Act (WMO). SAEs and a long-term increase of adverse events as a result of the interventions are not expected, since previous research has shown that treatment significantly reduces the odds of adverse events [77].

### Data management, data security, and quality control

The handling of personal data complies with the European General Data Protection Regulation (GDPR). Data are handled confidentially and data files are coded and separated from name, date of birth, and address data. Only the principal investigators have the key to this code and access to the source data. The CASTOR trial management software is used for this trial. Raw data will be kept for 5 years according to Dutch guidelines. A data management plan can be found at the Department of Psychosis research and Innovation, Parnassia Psychiatric Institute, The Hague, The Netherlands.

RoQua is hosted in data centres of the University of Groningen and operates in compliance with the NEN-ISO/IEC 27001 standard and the GDPR.

### Dissemination

The results of this study will be published in peer-reviewed journals and presented at national and international scientific conferences. Our international project team has the experience and network to ensure effective dissemination of the results to the national and international scientific community and into clinical practice. The sponsor will have no influence on the publication of the results.

## Discussion

The RE.PROCESS trial will be the first study to directly compare the effects of three types of TFT (CR, PE and EMDR) to TAU on PTSD symptoms in individuals with psychosis and PTSD. It is, in part, a replication study, aiming to validate the findings of previous RCTs on the effectiveness of PE, EMDR, and CR in people with psychosis [15–17, 21]. By targeting psychosis-related traumatic events in therapy when PTSD symptoms have sufficiently resolved, this study aims to address a broader concept of post-traumatic reactions that include psychosis. Secondary effects on clinical and functional outcomes, as well as cost-effectiveness, will be investigated providing a comprehensive investigation of the potential benefits of these therapies both directly after therapy and long term.

This study also aims to identify possibilities and challenges in implementing these therapies in routine clinical practice, although this is not a full implementation trial [83]. Additional benefits of the trial are that the therapists will be trained in all three TFTs and will deliver therapy as part of their clinical role in routine care. They will therefore be well placed to disseminate the TFTs, if found to be effective, in their services following completion of the trial.

## Trial status

Protocol version number and date: 1.13, 27 September 2022

Date of first enrolment: June 24, 2019 (current sample size: 103)

Date recruitment is expected to be completed: December 31, 2023

Expected date of study completion: December 31, 2025

Recruitment status: Recruiting

## Supplementary Information

**Additional file 1.** Model Consent Form.

**Additional file 2.** SPIRIT Checklist for *Trials*.

## Data Availability

The investigators will permit trial-related monitoring and audits by providing the Sponsor and METC direct access to source data and other documents as required. An anonymized version of the main outcome data and the corresponding statistical code will be available from the trial team on reasonable request after publication of the main results paper.

## Abbreviations

ASEX: Arizona Sexual Experience Scale
ASSIST: Alcohol, Smoking and Substance Involvement Screening Test
BDI-II: Beck Depression Inventory II
BRS: Brief Resilience Scale
CAPS-5: Clinician-Administered PTSD Scale for DSM-5
CBT: Cognitive behaviour therapy
CR: Cognitive restructuring
CRF-s: Counselor Rating Form – Short
DSM-5: Diagnostic and Statistical Manual of Mental Disorders-5th Edition
EMDR: Eye Movement Desensitization and Reprocessing
EQ-5d: EuroQol
ESSI: Enriched Social Support Instrument
GDPR: European General Data Protection Regulation
GEE: Generalized Estimating Equations
ICERs: Incremental cost-effectiveness ratios
ITQ: International Trauma Questionnaire
ITT: Intention-to-treat
LMM: Linear mixed models
METC: Medical Ethics Committee
PCL-5: PTSD Checklist for DSM-5
PE: Prolonged exposure
PSYRATS: Psychosis Symptom Rating Scales
PTCI: Posttraumatic Cognitions Inventory
PTSD: Post-traumatic stress disorder
QALYs: Quality-adjusted life years
QPR: Questionnaire about the Process of Recovery
RCT: Randomized controlled trial
R-GPTS: Revised Green et al. Paranoid Thought Scales
SAE: Serious adverse event
SAS: Sexual Autonomy Scale
SCID-5: Structured Clinical Interview for DSM-5
SPIRIT: Standard Protocol Items for Randomized Trials
SUD: Subjective Units of Distress
TAEQ TTIP: Adverse Events Questionnaire
TALE: Trauma And Life Events Checklist
TAU: Treatment as usual
TDSQ-s: Trait State Dissociation Questionnaire – short version
TFT: Trauma-focused treatment
TiC-P: Treatment Inventory of Costs in Patients with psychiatric disorders
VIS: Voice Impact Scale
VU: Vrije Universiteit Amsterdam
WAI-SR: Working Alliance Inventory – Short Form Revised
WL: Waiting list control group
WMO: Dutch Medical Research Involving Human Subjects Act
