# Supplementary material for: Trauma-focused therapies for post-traumatic stress in psychosis: study protocol for the RE.PROCESS randomized controlled trial
Source: Trials. 2022 Oct 5;23:851. doi: 10.1186/s13063-022-06808-6 (PMC9532824; doi:10.1186/s13063-022-06808-6)
Supplement: Supplementary file 1 — Additional file 1. Model Consent Form. [file 13063_2022_6808_MOESM1_ESM.docx]

**Additional file 1: Model Consent Form**

**Traumabehandeling bij mensen met psychose**

- Ik heb de informatiebrief gelezen. Ook kon ik vragen stellen. Mijn vragen zijn voldoende beantwoord. Ik had genoeg tijd om te beslissen of ik meedoe.
- Ik weet dat meedoen vrijwillig is. Ook weet ik dat ik op ieder moment kan beslissen om toch niet mee te doen of te stoppen met het onderzoek. Daarvoor hoef ik geen reden te geven.
- Ik geef toestemming voor het informeren van mijn regiebehandelaar dat ik meedoe aan dit onderzoek en deze te informeren over de uitkomsten van het inclusie-interview.
- Ik geef toestemming voor het opvragen van informatie bij mijn huidige behandelaar over mijn medicatie gebruik, duur van behandeling en gestelde diagnoses.
- Ik geef toestemming voor het maken van video-opnames van alle behandelsessies ten behoeve van supervisie en controle van kwaliteit van de behandeling.
- Ik geef toestemming voor het verzamelen en gebruiken van mijn gegevens voor de beantwoording van de onderzoeksvraag in dit onderzoek.
- Ik weet dat voor de controle van het onderzoek sommige mensen toegang tot al mijn gegevens kunnen krijgen. Die mensen staan vermeld in deze informatiebrief. Ik geef toestemming voor die inzage door deze personen.
- Ik geef □ **wel**

□ **geen**toestemming om mij na dit onderzoek opnieuw te benaderen voor een vervolgonderzoek.

- Ik wil meedoen aan dit onderzoek.

Naam proefpersoon:

Handtekening: Datum : __ / __ / __

-----------------------------------------------------------------------------------------------------------------

Ik verklaar dat ik deze proefpersoon volledig heb geïnformeerd over het genoemde onderzoek.

Als er tijdens het onderzoek informatie bekend wordt die de toestemming van de proefpersoon zou kunnen beïnvloeden, dan breng ik hem/haar daarvan tijdig op de hoogte.

Naam onderzoeker (of diens vertegenwoordiger):

Handtekening: Datum: __ / __ / __

-----------------------------------------------------------------------------------------------------------------

* Doorhalen wat niet van toepassing is.

De proefpersoon krijgt een volledige informatiebrief mee, samen met een getekende versie van het toestemmingsformulier.
